# First-trimester uterine rupture in a scarred uterus: a case report

**DOI:** 10.1093/jscr/rjaf617

**Published:** 2025-08-12

**Authors:** Charles Mawunyo Senaya, Francis Jojo Moses Kodzo Damalie, Emmanuel Nii Okai Okine, Bernard Kwabena Brewu

**Affiliations:** Directorate of Obstetrics and Gynecology, Komfo Anokye Teaching Hospital, P.O. Box KS 1934, Kumasi, Ghana; Department of Obstetrics and Gynecology, School of Medical Sciences, Kwame Nkrumah University of Science and Technology, PMB, Kumasi, Ghana; Directorate of Obstetrics and Gynecology, Komfo Anokye Teaching Hospital, P.O. Box KS 1934, Kumasi, Ghana; Department of Obstetrics and Gynecology, School of Medical Sciences, Kwame Nkrumah University of Science and Technology, PMB, Kumasi, Ghana; Directorate of Obstetrics and Gynecology, Komfo Anokye Teaching Hospital, P.O. Box KS 1934, Kumasi, Ghana; Directorate of Obstetrics and Gynecology, Komfo Anokye Teaching Hospital, P.O. Box KS 1934, Kumasi, Ghana

**Keywords:** uterine rupture, first-trimester complications, hemoperitoneum, scarred uterus, myomectomy, caesarean section scar

## Abstract

First-trimester uterine rupture is a rare but potentially fatal complication of pregnancy. It usually presents a diagnostic challenge because intrauterine gestation may still be visible on ultrasound even after uterine rupture with associated hemoperitoneum. The typical signs of uterine rupture, including abdominal tenderness, hemoperitoneum, and hemodynamic instability, become the guiding principle when considering a decision on emergency laparotomy. In patients with previous uterine scars, first-trimester ultrasonography should focus on excluding early placenta accreta spectrum, a condition that may lead to uterine rupture during the first trimester. A high index of suspicion is required in diagnosing a first-trimester uterine rupture.

## Introduction

Uterine rupture is a life-threatening complication of pregnancy. The majority of uterine ruptures occur in the third trimester and in women who have had previous uterine surgery. First-trimester uterine rupture is a rare event and can present a diagnostic challenge with consequent fatality [1].

The incidence of first-trimester uterine rupture is 0.07% globally, with a higher incidence of 1.3% reported in Africa [2]. The common causes of uterine scarring leading to uterine rupture are cesarean section, endometrial curettage, and myomectomy [3]. An interesting finding by Hamilton *et al*. that *in vitro* fertilization and embryo transfer (IVF-ET) could be an independent risk factor for first-trimester uterine rupture, irrespective of previous uterine surgery [4]. It has also been suggested that a placenta accreta spectrum (PAS) at this early stage of pregnancy may be the reason for the first-trimester uterine rupture [3].

The diagnosis of first-trimester uterine rupture may pose a challenge because the gestational sac may still be visualized *in utero* on ultrasound after the uterus has ruptured and hemoperitoneum has occurred.

The typical signs and symptoms of uterine rupture, such as hemoperitoneum and associated hemodynamic instability, severe abdominal pain, and vaginal bleeding, supersede the intact gestational sac *in utero* in deciding on laparotomy.

We report a case of first-trimester uterine rupture at 13 weeks following IVF conception in a patient with a history of myomectomy and two previous cesarean sections.

## Case presentation

Mrs DO, a 40-year-old G3P2 + 0 who conceived via IVF-ET, had a history of two cesarean sections and a myomectomy. She is also a known peptic ulcer disease patient. At 8 weeks gestation, she developed recurrent epigastric pain relieved initially by antacids and proton pump inhibitors but worsened over time, becoming unresponsive to medications.

At 13 weeks, she presented with worsening abdominal pain, dizziness, lightheadedness, and breathlessness. Physical examination revealed hypotension (BP 70/40 mmHg), tachycardia (pulse 108 bpm), severe pallor, and abdominal tenderness. A gynecological ultrasound scan showed fluid in the Morrison’s pouch but not in the pouch of Douglas (POD).

**Figure 1:**
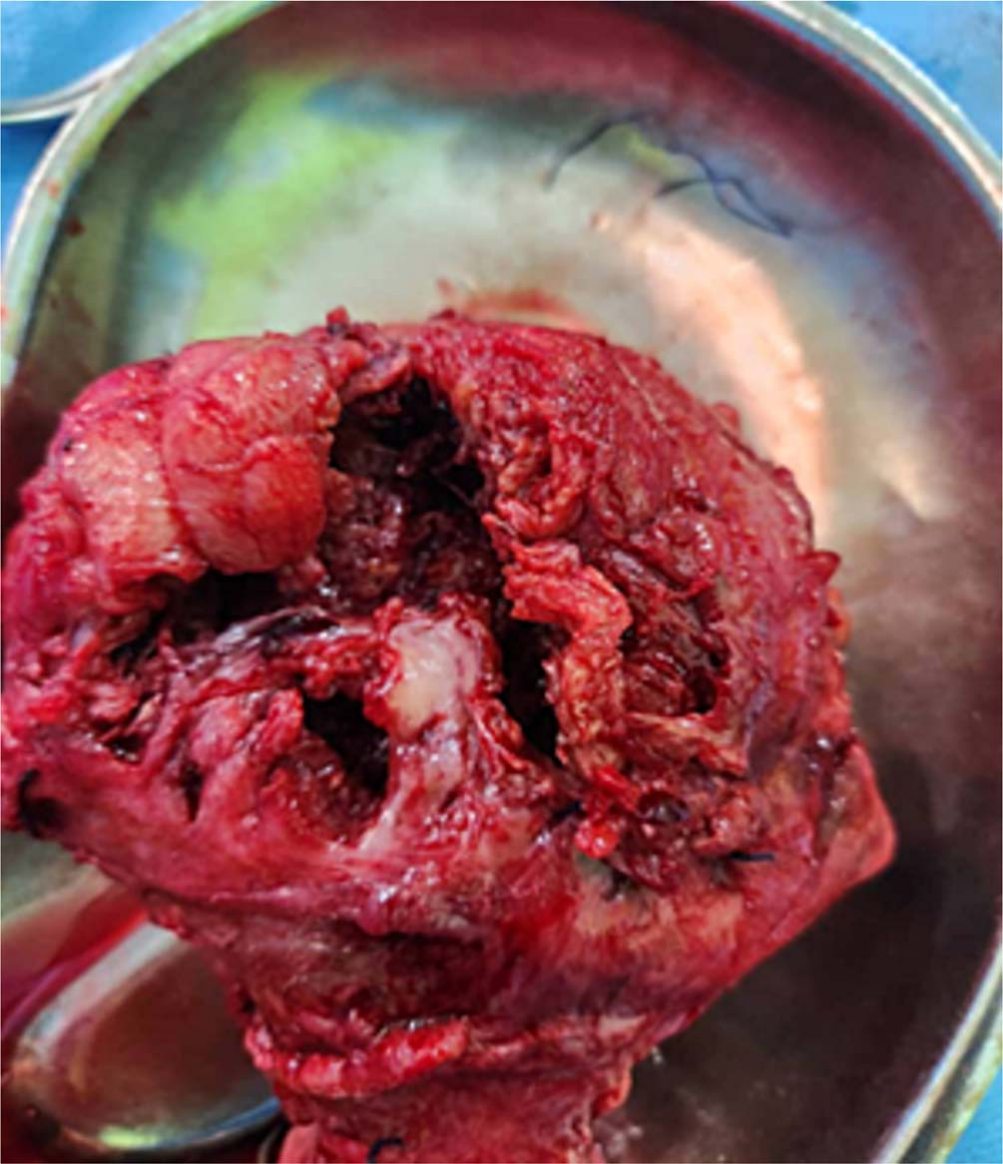
Extensive ragged rupture at the anterior aspect of the uterus.

**Figure 2:**
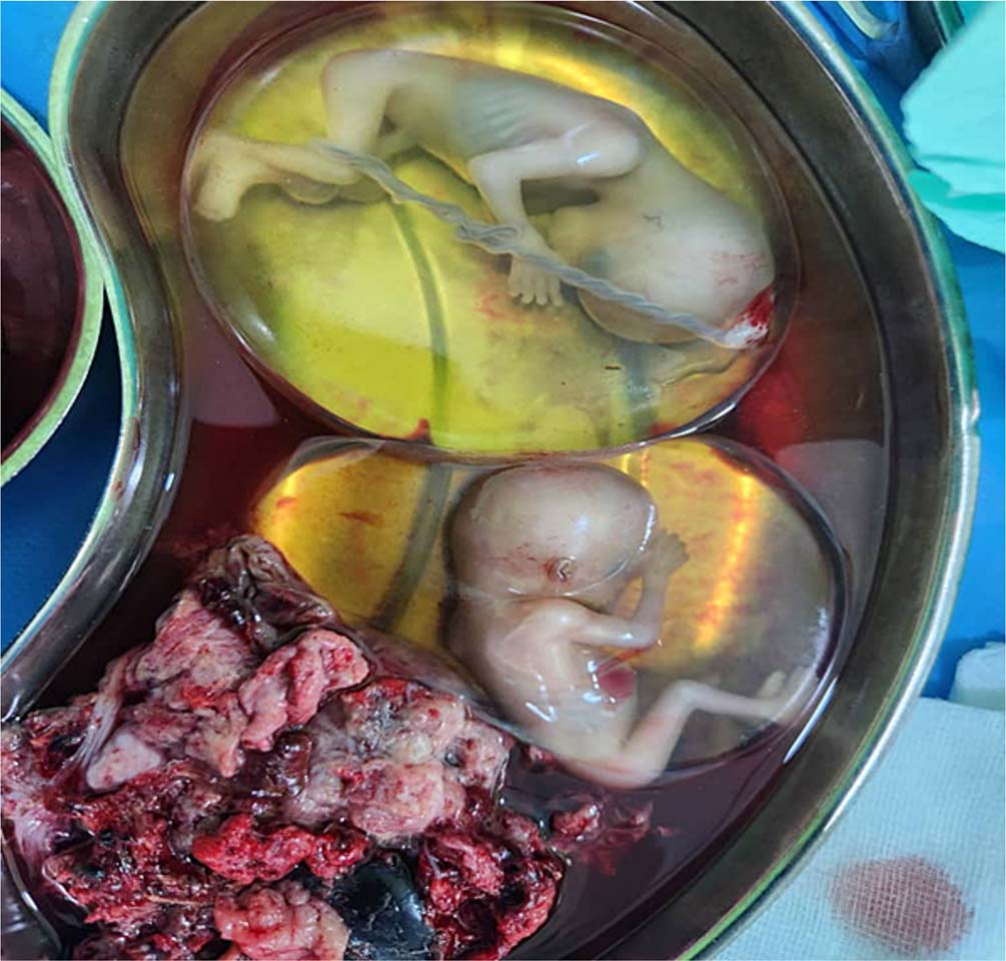
Fetuses in intact gestational sacs after removal from the ruptured uterus.

Emergency laparotomy revealed a uterine rupture at the anterior upper segment (Fig. 1) with about 2 l hemoperitoneum, an intact gestational sac (Fig. 2) *in utero*, and the placenta at the rupture site (Fig. 1). After appropriate counselling and in accordance with the couple’s informed request, a hysterectomy was performed during laparotomy. The patient was hemotransfused with four units of blood. Her postoperative recovery was uneventful, and she was discharged on postoperative Day 5.

## Discussion

Uterine rupture is strongly associated with uterine scarring. The common surgeries on the uterus that cause scarring include cesarean section, endometrial curettage, and myomectomy [3]. Our case had undergone myomectomy and two cesarean sections. Additionally, she conceived through IVF-ET, which has been implicated in uterine rupture, though the mechanism by which it increases the risk of uterine rupture remains unclear [5].

Uterine ruptures in the first trimester are attributed to either myometrial invasion by early PAS disorder or a scarred uterus [1]. In our case, the uterine rupture was toward the fundus, and the placenta was at the site of the rupture, making PAS the most likely cause. A Doppler ultrasound scanning to detect placenta invasion in the first trimester may aid in anticipation and early diagnosis of uterine rupture [6].

The abdominal pain in the first-trimester uterine rupture may be intermittent and insidious in onset with relatively hemodynamic stability, which may progress to suddenly severe generalized abdominal pain and shock, as occurred in our patient. The intermittent resolution of abdominal pain does not rule out the impending risk of uterine rupture.

The role of initial aggressive fluid resuscitation and ultrasound in aiding in the diagnosis is essential for the survival of the patient [7]. Abdominopelvic ultrasound findings may include the presence of hemoperitoneum in the Morrison’s pouch and or the pouch of Douglas. In first-trimester uterine rupture, the gestational sac may remain intact and *in utero*, as was in our case (Fig. 2). The presence of intact gestational sacs *in utero* and the absence of fluid in the POD may add to the challenge in making the diagnosis of uterine rupture.

Exploratory laparotomy is the recommended surgical option because the conversion from laparoscopy to laparotomy is high [3, 8]. Repair of the uterine defect is commonly carried out to retain fertility. However, in about a third of first-trimester uterine rupture, a hysterectomy may be required. The decision to opt for a hysterectomy should be individualized based on the patient’s history, presentation, intra-operative findings, the complexity of the repair, and the risk of future rupture [2]. These must be discussed with the patient before surgery. Our decision to undertake a hysterectomy was based on the complexity of the uterine rupture (Fig. 1), our presumed risk of future rupture, and the couple’s desire for a hysterectomy following the life-threatening experience.

## Conclusion

The occurrence of uterine rupture in the first trimester is a rare, life-threatening condition. Clinicians must have a high index of suspicion of uterine rupture when a patient with a scarred uterus presents with the typical signs of uterine rupture in the first trimester, even in the presence of an apparent intrauterine gestation. Early surgical intervention decreases the morbidity and mortality associated with first-trimester uterine rupture.
